# Advances in Understanding the Relationship between Sleep and Attention Deficit-Hyperactivity Disorder (ADHD)

**DOI:** 10.3390/jcm8101737

**Published:** 2019-10-19

**Authors:** Serena Scarpelli, Maurizio Gorgoni, Aurora D’Atri, Flaminia Reda, Luigi De Gennaro

**Affiliations:** Department of Psychology, University of Rome “Sapienza”, Rome 00185, Italy; serena.scarpelli@uniroma1.it (S.S.); maurizio.gorgoni@uniroma1.it (M.G.); aurora.datri@gmail.com (A.D.); flaminia.reda@uniroma1.it (F.R.)

**Keywords:** ADHD, sleep, PSG, macrostructure, microstructure, theta, slow wave activity, EEG, memory, cognition

## Abstract

Starting from the consolidated relationship between sleep and cognition, we reviewed the available literature on the association between Attention Deficit-Hyperactivity Disorder (ADHD) and sleep. This review analyzes the macrostructural and microstructural sleep features, following the Preferred Reporting Items for Systematic Reviews and Meta-Analyses criteria (PRISMA). We included the polysomnographic studies published in the last 15 years. The results of macrostructural parameters are mixed. Almost half of the 18 selected investigations did not find differences between sleep architecture of children with ADHD and controls. Five studies observed that children with ADHD show a longer Rapid Eye Movement (REM) sleep duration than controls. Eight studies included microstructural measures. Remarkable alterations in sleep microstructure of ADHD are related to slow wave activity (SWA) and theta oscillations, respectively, during Non-REM (NREM) and REM sleep. Specifically, some studies found higher SWA in the ADHD group than controls. Similarly, higher theta activity appears to be detrimental for memory performance and inhibitory control in ADHD. These patterns could be interpreted as a maturational delay in ADHD. Also, the increased amount of these activities would be consistent with the hypothesis that the poor sleep could imply a chronic sleep deprivation in children with ADHD, which in turn could affect their cognitive functioning.

## 1. Introduction

Attention Deficit-Hyperactivity disorder (ADHD) is one of the most common early childhood disorders, classified into three subtypes: predominantly inattentive, hyperactive-impulsive, and a combination of these two subtypes [1]. The prevalence of ADHD ranges from 5 to 7% and is more frequent in males [2].

Children with ADHD show heterogeneous symptoms, e.g., aggressiveness, inappropriate social conduct, and impaired academic functioning, due to a compromised inhibitory control [3,4]. It is well known that the neurobiological mechanisms underlying ADHD pathophysiology include some neurochemical agents, especially dopamine and noradrenaline [5].

A high percentage of subjects with ADHD (59–87%) reports psychiatric comorbidities, such as learning disabilities (15–25%), language disorders (30–35%), mood and emotional disorders (15–25%), motor coordination deficits (60%), and conduct disorders (20%) [5,6].

Sleep alterations are also significantly observed in 25–55% of children with ADHD compared to 7% among healthy individuals [7]. Subjective parent reports referred to sleep initiation problems or sleep fragmentation, with several night awakenings of their children [7]. Objective measures also revealed that Sleep-Disordered Breathing (SDB), Periodic Leg Movements during Sleep (PLMS) and Restless Leg Syndrome are strongly related to ADHD [8]. Excessive daytime sleepiness in children with ADHD represents a direct consequence of these sleep disturbances [9]. Besides, subjects with ADHD frequently suffer from the circadian-rhythm disorder and idiopathic sleep-onset insomnia [10]. These disturbances may be due to a delayed endogenous circadian pacemaker, as demonstrated by the alterations observed in the nocturnal pattern of melatonin secretion [10]. Difficulties in sleep offset are also reported in children with ADHD [11]. 

Starting from these observations, it can be hypothesized that sleep architecture (i.e., macrostructure) of children with ADHD shows significant changes compared to healthy individuals, especially concerning sleep onset latency, rate of awakenings, wakefulness after sleep onset, and other measures of sleep fragmentation. However, it is still unclear whether the alterations in sleep architecture in ADHD are only related to full-blown comorbid sleep disorders or whether the abnormalities in polysomnographic (PSG) measures are actually markers/symptoms of ADHD. 

Interestingly, an overlap has been observed between neurocognitive deficits characterizing children with ADHD and those affecting healthy people with a poor sleep quality [12]. Indeed, sleep problems in healthy individuals impact on inhibitory control and enhance their impulsivity [13]. On the one hand, sleep alterations may induce ADHD-like manifestations, and, on the other hand, altered sleep architecture could exacerbate ADHD symptoms [7].

In the last decades, several findings underlined that sleep features are associated with cognitive functioning [14]. In this view, early studies showed that poor sleep quality associated with SDB is related to low performance on the Verbal intelligence quotient (IQ) in subjects with ADHD [15]. Also, a reduced amount of Rapid Eye Movement (REM) sleep has been linked with impaired language, visuo-spatial, attention/executive functioning, and memory processes in children with ADHD [16], while an increased REM percentage is negatively correlated with IQ [17].

Beyond this, it is worth noting that some electroencephalographic (EEG) oscillations during sleep (e.g., sleep spindles, slow waves, and theta activity) seem to play a crucial role in neural plasticity and learning processes [18,19]. Specifically, several findings revealed that both slow wave activity (SWA) and theta oscillations show quantitative and topographical changes during development, which parallel the brain maturation [14]. Considering that some neuroanatomical studies highlighted a delay in cortical maturation of children with ADHD [20], we suggest that a better understanding of the relationship between sleep alterations and this neurodevelopmental disorder could be helpful to design protocols aimed to enhance sleep quality and to manipulate sleep EEG oscillations to ameliorate ADHD symptoms.

In light of the above, we reviewed the available literature of the last 15 years on the association between ADHD and sleep, focusing on the macrostructural and microstructural features of sleep. We aim to provide a comprehensive background that underlines the strengths and limitations of the current knowledge, to track future researches and perspectives.

## 2. Materials and Methods

### 2.1. Search Strategy 

This systematic review was performed following PRISMA (Preferred Reporting Items for Systematic Reviews and Meta-Analyses) criteria [21]. A search was conducted on two electronic databases: PubMed and Scopus, entering the following search terms in English: “ADHD” OR “attention deficit” OR “hyperactivity” AND “sleep” AND “polysomnography”. Also, the following search string was considered: “ADHD” OR “attention deficit” OR “hyperactivity” AND “sleep” AND “EEG”. Search fields were title, abstract and keywords. Only quantitative research/original articles published in the last 15 years were further analyzed. Eligible articles were selected through a multi-step procedure (title reading, abstract and full-text assessment) by 2 independent expert researchers. Then, the literature search was completed with a manual search, reviewing the references included in the selected articles and the citations that they had received.

### 2.2. Selection Criteria

Articles available from 2004 until May 2019 were selected if they met the following criteria: (1) Inclusion of children with ADHD, according to the criteria of the Diagnostic and Statistical Manual of mental disorders (DSM) or any other diagnostic manual; (2) absence of intellectual disabilities (IQ < 70); (3) comparative studies in which the control group was composed of children without ADHD; (4) focus on PSG recordings of a night of sleep with macrostructural (and/or microstructural) measures were reported, assessing differences in sleep between children with and without ADHD; (5) peer-reviewed articles (not just abstracts or conference papers). Reviews, meta-analyses and papers in non-English languages were excluded.

**Figure 1:**
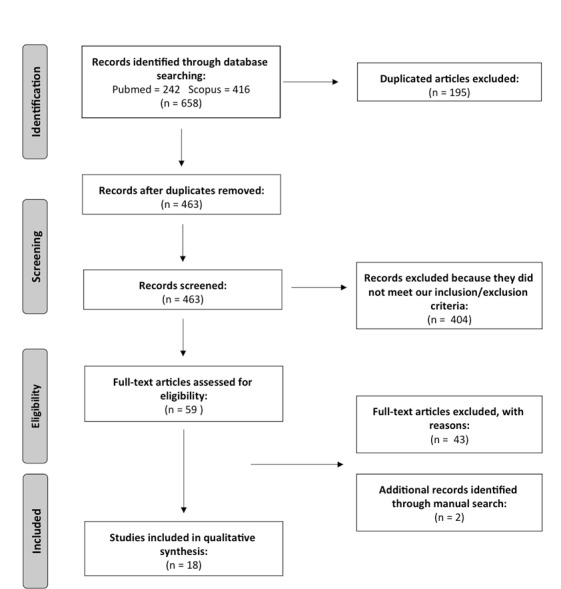
The Preferred Reporting Items for Systematic Reviews and Meta-Analyses (PRISMA) workflow. Eighteen studies were considered for this systematic review.

A first selection was performed by filtering duplicates and, subsequently, a title and abstract screening was conducted. All potentially relevant articles were then independently reviewed and assessed for their eligibility. Studies which included ADHD samples, but whose primary focus was on other disturbances, were also excluded. We considered only studies that fulfilled the inclusion criteria previously described and addressed the question on the relation between PSG/EEG measures and ADHD and/or reported microstructural sleep measures. Any disagreement between the reviewers was resolved through a consensus session with a third reviewer. Figure 1 shows the flowchart of the article selection.

## 3. Results

At the end of the multi-step process, 18 articles [22,23,24,25,26,27,28,29,30,31,32,33,34,35,36,37,38,39] were included in the systematic-review. The results were grouped in the following section, on the basis of the sleep measures obtained in each study:

(a) Macrostructural pattern (18 articles) [22,23,24,25,26,27,28,29,30,31,32,33,34,35,36,37,38,39];
(b) Microstructural pattern (8 articles) [23,26,30,31,33,34,36,37].

Table 1 summarizes the characteristics and main results of the reviewed studies. Table S1 (Supplementary Materials) reports the list of abbreviations and their definitions.

Only data on PSG recordings during nighttime were considered (e.g., excluded Multiple Sleep Latency Test, MSLT or daytime naps). In the case of longitudinal protocols, only the first PSG assessment was taken into account.

### 3.1. Macrostructural Pattern

All of the 18 selected studies [22,23,24,25,26,27,28,29,30,31,32,33,34,35,36,37,38,39] reported at least the following sleep measures: sleep onset latency (SOL); stages duration (stage 1,2,3,4 or SWS, REM sleep); total bedtime (TBT); total sleep time (TST); sleep efficiency (SE); wakefulness after sleep onset (WASO). Some investigations also reported the sleep latency of other sleep stages (REM; SWS); sleep period time (SPT) [22,23]; stage shift (SS) [23] and the number of cycles [22,35].

Eight out of 18 studies did not report any difference in sleep macrostructural measures between children with ADHD and healthy controls (HC) [25,26,29,30,31,32,37,38].

Concerning TST and TBT, mixed results were observed. On the one hand, TBT seems to be longer in children with ADHD, as compared to HC [22,29]. On the other hand, shorter TBT was described in the ADHD group [23,36].

Multiple recordings revealed that TST is lower in the ADHD than the HC group [23,24,35], while only one study reported the opposite finding [29]. Instead, a study reported longer SPT [22] and another lower SPT in the ADHD compared with the HC group [23].

Reduced SE was reported in the ADHD compared with the HC group [27] and, consistently, several measures of sleep fragmentation were observed: a higher rate of SS [23] and a higher number of sleep cycles in the ADHD groups [22,35].

Concerning REM sleep, only a study reported a lower REM sleep percentage in the ADHD group [24], while others observed a higher REM sleep duration [22,27,29,34,35], as compared to the control group. Also, a shorter REM sleep latency was found in the ADHD group [29]. 

Some results revealed alterations of NREM sleep in ADHD, pointing to a reduction of SWS in children with ADHD [35,39]. A lower duration of stage 1 was also detected in the ADHD than in the control group [33,35].

Nine studies included an ADHD sample without comorbidities [25,29,30,32,33,34,36,37,39]. Virring et al. [32] reported analyses also on ADHD without comorbidities, showing only longer SOL, compared with controls. Consistently, longer SOL in children with ADHD than HC was found by Prehn.-Kristensen et al. [27].

Eleven studies were carried out with an adaptation night in a laboratory setting [22,23,25,26,27,29,30,31,34,36,38], while 4 studies used home recordings [24,28,32,35].

Interestingly, PSG parameters were studied in relation with cognitive performance in 6 studies [26,27,31,36,37,38]. In this respect, sleep-related gains in reaction times to procedural memory tasks were positively correlated with the percentage of stage 4 and with REM sleep density in the ADHD group [26]. The same study also found a positive correlation between the amount of NREM sleep and the sleep-associated declarative memory consolidation in HC, while no relation between macrostructural measures and performance was found in children with ADHD [27]. Wiesner et al. [38] revealed no significant correlation between the consolidation of rewarded behavior and sleep measures. The other studies administering cognitive tasks have not reported correlational analyses between the children’s performance and macrostructural measures: the authors provided correlational analyses exclusively considering microstructural measures (see next paragraph) [31,36,37]. 

### 3.2. Microstructural Pattern

Eight out of 18 studies reported microstructural sleep measures [23,27,30,31,33,34,36,37].

#### 3.2.1. NREM Sleep

Microstructural features of sleep in children with ADHD were firstly investigated by Miano et al. [23] through CAP analysis. The main result of their study was a reduction of CAP A1 phase (i.e., synchronized EEG sleep pattern with sequences of K complexes and delta bursts) during light sleep (i.e., stage 1 and stage 2 NREM), but no differences were detected in other CAP subtypes. These results were recently confirmed [34], while another investigation did not replicate them [30].

Moreover, the quantitative EEG analysis of sleep revealed some differences between children with ADHD and HC, providing information on the topographic distribution of sleep EEG activity (e.g., [33,37]) and its relation with neurobehavioral and/or cognitive domains [27,31,36,37]. 

Ringli et al. [33], using high-density EEG (128 channels), observed a higher SWA during stage 2 and stage 3 over the central area in the ADHD group, with a maximum placed posteriorly as compared with HC. The differences between the two groups were maintained across the entire night. Moreover, they did not find the typical decrease of SWA across consecutive sleep cycles, when analyzing the homeostatic decay in the motor area [33]. The waking EEG activity of children with ADHD and the percentage of SWA were not correlated, contrary to the expectation of the authors based on the “local sleep hypothesis” (i.e., local use-dependent view, [40]).

Other studies investigated EEG power during NREM sleep without confirming the higher amount of SWA in the ADHD group [27,31,36,37]. However, some studies assessed SWA in relation to memory processes [27,31]. Children with ADHD, as compared to HC, showed a lower overnight improvement of recognition accuracy at the picture recognition task (i.e., participants were asked to rate their emotional state while fixing a set of emotional and neutral picture; then memory were assessed by an immediate and delayed recognition session) [27]. Specifically, while this gain was positively correlated to the amount of SWA in the first sleep cycle, no correlation was observed for children with ADHD. Partly in line with these results, an investigation from the same research group found that the overnight gain at a memory task (i.e., picture recognition) was positively correlated with slow oscillations of sleep EEG (i.e., <1 Hz activity) in the healthy group including children and adults [31]. This study also showed that the compromised memory performance in the ADHD group was negatively correlated to the slow oscillations during SWS [31].

The EEG theta activity during NREM sleep was not investigated in the absence of any neurocognitive task. Prehn-Kristensen et al. [27] did not find any significant difference between children with ADHD and HC in this frequency band, and no correlation with memory performance was significant [27]. More recently, Cremone et al. [37] observed that the theta activity in NREM sleep did not differ between the ADHD and the control group. In both samples, the inhibitory control was tested and no correlation was observed between theta activity and the overnight gain at the Go/noGo task [37]. 

Both sigma and theta activity during NREM sleep were examined in relation to motor learning with the Motor Sequence Task (MST) [36] The theta power in stage 2 was mildly higher in children with ADHD than HC, but no correlation was found with the motor task. Concerning the sigma spindle-related activity, precision at the motor task was positively correlated with slow-frequency sigma power in the ADHD group, but no correlation with the sleep-related gain was observed. It is worth noting that performance of the ADHD group did not differ from that of HC. However, the slow and fast sigma activity during stage 2 was reduced in children with ADHD compared to controls [36]. 

When sigma power [31] and spindle density [27] were assessed in children engaged in the episodic/emotional memory task, no differences were found between the ADHD and control group. In addition, the sigma/spindle activity and overnight gain were not correlated [27,31].

Concerning alpha EEG activity, no difference was found between children with ADHD and HC when examined in association with declarative/emotional memory [27].

#### 3.2.2. REM Sleep

As mentioned for NREM sleep, theta activity was assessed only in protocols involving specific learning/memory tasks [27,31,37]. On the one hand, the theta activity did not show correlation with the gain at the Picture Recognition Task in children with ADHD and HC [27], and no differences were found in the theta power between children with ADHD and children with typical development [27,31]. On the other hand, the memory performance in the ADHD group was negatively correlated to the theta activity, similarly to what has been observed for the amount of SWA [31]. Healthy subjects including children and adults, differently from children with ADHD, revealed a positive correlation between the overnight gain on a memory task and theta activity [27].

Finally, theta power was examined in relation to inhibitory control as measured by a Go/noGo task, tested before and after sleep [37]. This study reported that children with ADHD had a higher theta power during REM sleep than HC. Moreover, HC showed a greater accuracy at the inhibitory control task after sleep (vs. performance before sleep), and this improvement was related to theta power. Conversely, children with ADHD did not report any sleep-related gain [37].

No remarkable finding on the alpha activity was reported [27].

## 4. Discussion 

We reviewed for the first time the studies concerning the relation between macrostructural and/or microstructural features of sleep and ADHD, considering the investigations of the last 15 years. 

### 4.1. Macrostructural Pattern

The results concerning the macrostructural parameters are quite conflicting, and drawing a coherent framework is challenging. Almost half of the selected studies did not find differences in the sleep architecture of children with ADHD, compared to those with typical development [25,26,28,30,31,32,37,38]. 

The investigations revealing significant differences between the ADHD and HC groups demonstrated that the clinical samplea are characterized by poorer sleep than controls. Indeed, the longer SOL [27,35], the shorter TST [23,24,35], along with the reduced SE [27] and the indices of faster transition of stages/cycles (e.g., a higher rate of SS and a greater number of sleep cycles) [22,23,35] reflect -to some extent- the sleep dysregulation in ADHD. On the one hand, the assumption that children with ADHD have an altered sleep architecture is consistent with the subjective parent reports, mentioning a delayed sleep onset or a sleep fragmentation with several night awakenings [7]. On the other hand, the sleep difficulties reported by parents appear to be significantly higher as compared to objective measures [8,39]. The gap between subjective and objective measures may be ascribed to other factors that cannot be revealed by the PSG recordings. The subjective reports of increased SOL—confirmed only by some investigations using objective measures (e.g., [27,35])—may be explained by “behavioral” sleep problems occurring *before* bedtime [7]. In this regard, the bedtime resistance could depend on multiple factors: (a) inadequate parenting; (b) Inadequate pre-sleep bed routines and/or sleep environment; (c) pharmacotherapy side-effects. This disturbance in ADHD children is often interpreted as oppositional behavior, a significant source of distress for parents [41]. In light of the above, it is fair to assume that subjective reports could overestimate sleep problems in ADHD, reflecting parental concerns.

NREM sleep architecture did not show specific and consistent differences between children with ADHD and HC [22,23,24,25,26,28,29,30,32,34,36,37,38]. We have to underline that a lower SWS has been found in an ADHD sample characterized by a higher rate of comorbidities [35] or when a higher apnea-hypopnea index was detected [39]. In this respect, a strong relationship between SDB and ADHD behavior was found (e.g., [42]), likely inducing the disruption of sleep homeostasis and less deep sleep [43]. Moreover, as previously mentioned, the exclusion of subjects with externalizing and internalizing problems from the analysis abolished all differences in NREM sleep [35]. 

Although no other differences in SWS were found, it should be underlined that both stage 4 amount and REM sleep density were correlated with the improvement in motor skills performance after sleep in children with ADHD, as assessed by Prehn-Kristensen et al. [26]. This study suggested a possible beneficial role of sleep on tasks measuring procedural memory in ADHD, that –instead- showed compromised performance during the waking state [26].

REM sleep abnormalities seem to be widespread among children with ADHD, consistently with previous meta-analysis [44]. REM sleep alterations could be related to the dysfunctional reward learning, one of the core deficits of ADHD, due to the dopamine hypofunction [45].

Specifically, several findings highlighted that REM sleep duration is longer in children with ADHD compared to controls [22,27,29,34,35], and only Gruber et al. [24] found a reduced amount of REM sleep in ADHD. It is worth noting that this latter study included patients with PLMS that may induce REM sleep instability, contributing to its shorter duration [46]. 

Interestingly, the higher REM sleep percentage in the ADHD group was found along with a higher number of sleep cycles in two studies [22,35]. As already suggested, the increased number of sleep cycles may result in a faster transition to REM sleep, contributing to the increased REM sleep amount [22]. Once again, the presence of comorbidities could have affected these results. 

### 4.2. Microstructural Pattern

Concerning the eight studies reporting sleep microstructural features in the ADHD group, the results are not homogenous [23,27,30,31,33,34,36,37].

Findings on spindle activity are still scarce [27,31,36] and very difficult to interpret. In particular, some methodological differences in the spindle analysis should be taken into account: (a) Saletin et al. [36] did not provide a specific spindle detection, analyzing the whole sigma band; (b) some protocols [27,31,36] did not include parietal derivations, where the spindles typically show their maximum [47].

The most relevant alterations in sleep microstructure of ADHD are related to SWA and theta activity during NREM and REM sleep, respectively. 

Firstly, starting from the perspective that SWA has an age-dependent shift along the postero-anterior axis from 2 years of age to adolescence [48,49], some findings point to an alteration of SWA in children with ADHD [33]. Also, during a typical development the distribution of SWA mirrors the cortical maturation changes, characterized by an increment in the first years of life with a peak in puberty and a decrease during adolescence [48,49]. In this vein, the higher SWA over the central region in ADHD—observed by Ringli et al. [33]—may represent a sign of developmental delay. The existence of a maturational delay in ADHD has also been proposed on the basis of the improvement of ADHD symptoms during growth (e.g., [20,50]). Consistently, neuroimaging studies showed that the onset of grey matter maturation in ADHD is delayed by around 3 years compared to HC, and the remission of ADHD symptoms appears associated with cortical normalization [50]. However, other investigations did not confirm this finding [27,31,36,37], likely because of the different age brackets of the included sample [34] and the lower number of EEG channels considered [27,31]. Moreover, it should be emphasized that the EEG measures in most of the studies have been recorded in combination with specific cognitive tasks [27,31,36,37], that may have induced changes in sleep EEG oscillations [14]. When the memory domain was assessed, the expected relation between SWA and performance was not observed in the ADHD group. It could be hypothesized that impairment in the sleep-dependent memory processes is due to compromised functioning over the frontal region, where the slow oscillations originate [51].

Since CAP A1 subtypes are involved in the buildup of NREM deep sleep, the finding of a reduced A1 rate during light sleep in children with ADHD [22,31] is partially not coherent with the higher SWA revealed by Ringli et al. [33]. It should be mentioned that the higher slow/delta activity in the ADHD group could represent a microstructural index of chronic partial sleep deprivation due to an arousal dysregulation in this neurodevelopmental disorder [23,34]. However, we point to that the CAP analysis does not provide direct information about the specific CAP components (e.g., delta bursts and K-complexes for A1). Hence, CAP A1 should be interpreted only cautiously in terms of high EEG synchronization, which is conventionally measured by the quantitative EEG analysis.

Concerning REM sleep microstructural features, we highlighted that studies were focused only on the theta activity [27,31,37]. Along with SWA, the theta oscillations also mirror changes in cortical plasticity and brain maturation [14,49]. Specifically, the theta activity in HC declines earlier during development than SWA, and it appears to be independent from the sleep stage [3,27]. In this view, the findings by Cremone et al. [37] on the higher theta activity in an ADHD sample, characterized by a lower mean age (6.7 vs. 11.9), could represent a sign of maturational delay, as already suggested for SWA [33].

Moreover, the abnormal theta functioning in children with ADHD is related to a bad cognitive performance [31,37]. A higher theta activity is associated with an impaired inhibitory control in ADHD [37]. Similarly, concerning the memory consolidation at the picture recognition task, the greater theta activity in the ADHD group, along with the amount of SWA, is linked to a weaker performance [31]. It should be considered that the picture recognition task included emotional stimuli from the International Affective Picture System, providing –to some extent- a measure of emotional memory [31].

Several EEG waking studies showed that the theta activity has a pivotal role in ADHD functioning [52]. In particular, children with ADHD show an increased absolute theta power, often associated with a decreased beta power. This leads to an increased theta/beta ratio, recognized as a neurophysiological marker for helping to diagnose ADHD [52]. These findings could be conceptualized in terms of “cortical hypoarousal” and of unstable vigilance regulation [53]. Moreover, waking theta EEG activity is related to inhibitory control in healthy children [54].

In light of these considerations, we can speculate that the altered theta functioning could represent a biomarker of ADHD during sleep as well as during waking state. Considering that theta oscillations in healthy individuals are usually associated to some cognitive functions when they occur during REM sleep [31,55,56,57], it could be hypothesized that theta activity has a cut-off level which, if exceeded, leads to a compromised performance [31,37]. 

### 4.3. Limitations

The reviewed studies assessing macrostructural and microstructural sleep features are affected by several confounding variables, and some methodological considerations are needed: (a) the occurrence of sleep disorders [22] or psychiatric disturbances [22,23,35] could strongly affect the sleep structure of children [58]. Indeed, a high percentage of ADHD samples included subjects with oppositional defiant/conduct disorders [22,26,27,28,31,35,38], learning disabilities [22,23,38] or internalizing comorbidities [22,23,35], without tracking any linear relation among these disorders and sleep patterns; (b) the age range of the considered sample can explain some discrepancies on sleep measures (e.g., [33] vs. [36]), since the sleep microstructure significantly changes during development co-varying with brain maturation [48,49]; (c) the different subtype of ADHD could impact on sleep pattern [59]. In particular, the prevalence of the combined subtype in most of the reviewed studies [22,23,24,25,28,29,30,33,34,35,37,38,39] did not allow to draw any conclusion on the differences between subtypes; (d) the children’s medications should be also assessed, considering the interaction between stimulants and sleep [60]. All considered studies reported that subjects stopped the medications during the experimental session. However the long-term stimulant effects could be significant. Moreover, some moderating factors could impact on the effects of medications (e.g., Body Mass Index/weight, length and time on stimulants, gender [60]), and these factors should be taken under control; (e) the presence/absence of an adaptation night and the experimental setting (laboratory or home recordings) may impact on sleep fragmentation [29]; (f) the inclusion of borderline cognitive participants (e.g., [22,35,39]) should be controlled, considering that some studies revealed that borderline intellectual functioning could impact on sleep features [61]; (g) the absence—in certain studies—of baseline sleep recordings without a task administration during the evening represents a substantial limitation. Indeed, the differences between ADHD groups and controls were found only when the correlational analyses on performance were considered and not when microstructural features were directly compared between the clinical and control groups [27,31,36,37].

Besides, concerning microstructural measures, specific methodological limitations should be underlined. Firstly, the studies -with some exceptions [33,37]—included a small number of EEG channels and analyses on CAP parameters are not effective in providing information on the regional/topographical distribution of the observed phenomena. Moreover, although the relationship between the frontal theta rhythm and cognitive functioning is well-established in wakefulness (for review, see [62]) and sleep [55,56], we underline that the conventional quantitative EEG analysis (i.e., by using Fast Fourier Transform routines) used in the reviewed studied [27,31,33,36,37] is mainly designed for stationary signals [63] and could fail in the detection of oscillatory activity. We point to that the rhythmic/oscillatory theta activity should be distinguished from the non-rhythmic theta waves identified in relation to hypoarousal and sleepiness during the waking state (i.e., local sleep; [40,64]). In this regard, it has been recently demonstrated, by using a technique for detecting bursts of theta activity, that the theta activity associated with prolonged wakefulness is expressed by "isolated" waves and not by rhythmic oscillations [64]. In this vein, recent studies introduced a new method (i.e., Better OSCillation [65]) to successfully discriminate theta oscillations from background signals [63], revealing that rhythmic theta is related to mental/cognitive sleep activity [66,67]. Taken into account these considerations, a protocol including this specific analysis/detection may shed light on the nature of the abnormal theta activity in ADHD, providing differentiation between theta oscillations (related to cognitive encoding) and non-rhythmic activity (related to the hypoarousability).

## 5. Conclusions

To sum up, consistently with a recent meta-analysis [57], we emphasized that the sleep architecture of children with ADHD reported only slight differences compared to HC. The results are mixed, and the available findings did not provide a clear and comprehensive framework on the issue. Conversely, some microstructural EEG signatures, albeit heterogeneous, account for the specific link between sleep pattern and the domains of cognitive or neurobehavioral functioning. Specifically, the microstructural features showed that both SWA and theta oscillations are altered in children with ADHD, while evidence on other activities is still scarce.

The dysfunctional modulation of these—predominantly fronto-central—activities may represent the expression of a general deficit in the interplay of the fronto-limbic circuits in ADHD. Consistently, neuroimaging data revealed dysfunctions over the frontal region, the striatum and the cerebellum and, not surprisingly, neuropsychological deficits may arise from reduced brain functions [50].

Another perspective suggested that greater SWA and theta activity could be a sign of higher sleep pressure in children with ADHD [68]. The increased amount of these activities would be consistent with the hypothesis that the poor sleep could imply a sort of chronic sleep deprivation in children with ADHD [23], which in turn could affect their cognitive functioning. This appears to be in line also with the fact that sleep deprivation can impact on the prefrontal cortex, involved in several cognitive processes [12]. 

Although the empirical evidence is still preliminary, we propose that the detection of sleep EEG anomalies in children with ADHD could represent a starting point to provide a target to develop future interventions. In this respect, recent studies revealed that the slow oscillating (0.75 Hz) transcranial direct current stimulation [69] during NREM sleep increased the slow frontal oscillations in children with ADHD, positively contributing to declarative memory performance [50] as well as behavioral inhibition [70]. Based on these promising results [69,70], it would be interesting to design protocols aimed to ameliorate sleep and cognitive/ behavioral functioning in children with ADHD using tools that can modulate the altered sleep signatures. However, further investigations are necessary to provide useful insights at this issue.

## Figures and Tables

**Table 1:** Summary of characteristics of included studies.

Authors.	Sample Size (Sex; Mean Age)	ADHD Subtypes	IQ and Comorbidities	Medications	PSG Recording (Setting; EEG Channels)	Sleep Measures	Main Results
**Kirov et al. [22]**	17 ADHD (all M; 11.2 ± 2.0) vs. 17 HC (all M; 11.2 ± 2.3)	All combined	Full-scale IQ ≥ 80 ADHD: 12 with dyslexia; 3 with conduct disorder; 1 with panic disorder; 1 with nocturnal enuresis	11 ADHD stopped medications at least 3 days previous the experimental session	Laboratory recording with adaptation night; 1 EEG channel (C3)	Macrostructure	Children with ADHD had higher TBT, longer SPT, longer REM sleep duration and more sleep cycles than HC.
**Miano et al. [23]**	20 ADHD (18 M; 9.3 range: 6–13) vs. 20 HC (18 M; 9.3 range: 6–13)	2 inattentive 18 combined	Full-scale IQ ≥ 70 ADHD: 10 with learning disabilities; 4 with mild neurological signs; 2 with language disorder; 4 with psychiatric comorbidities	None	Laboratory recording with adaptation night; EEG channels not specified (at least 8 electrodes)	Macrostructure CAP parameters	Children with ADHD had lower TST, SPT, TBT and higher rate of SS than HC. Children with ADHD had lower total CAP rates and lower CAP rates during stage 2 than HC. Children with ADHD had lower CAP sequences and a reduced total A1 index in stages 1 and 2.
**Gruber et al. [24]**	15 ADHD (10 M; 8.45 ± 1.39) vs. 23 HC (13 M; 8.58 ± 1.27)	1 hyperactive 2 inattentive 12 combined	Full-scale IQ ≥ 80 ADHD: 2 with PLMD HC: 2 with PLMD	ADHD stopped medications at least 7 days previous the experimental session	Home recording; 8 EEG channels (F3, F4, C3, C4, P3, P4, O1, O2)	Macrostructure	Children with ADHD had lower TST, lower percentage of REM sleep than HC.
**Prihodova et al. [25]**	31 ADHD (26 M; 9.3 ± 1.7) vs. 26 HC (22 M; 9.2 ± 1.5)	4 inattentive 27 combined	Full-scale IQ ≥ 80 None	None	Laboratory recording with adaptation night; 4 EEG channels (F4–C4, C4–P4, F3–C3, C3–P3, C4–A1, C3–A2)	Macrostructure	No significant differences were found on sleep parameters between groups.
**Prehn-Kristensen et al. [26]**	16 ADHD (not provided, 10.6 ± 0.88) vs. 16 HC (not provided, 11.00 ± 0.99)	8 inattentive 8 combined	full-scale IQ ≥ 85 ADHD: 4 with ODD.	12 ADHD stopped medications 2 days previous the experimental session	Laboratory recording with adaptation night; 2 EEG channels (C3, C4)	Macrostructure	No significant differences were found on sleep parameters between groups. A sleep-associated gain in reaction times of procedural memory task was positively correlated with the amount of stage 4 and REM sleep density in ADHD group.
**Prehn-Kristensen et al. [27] **	12 ADHD (all M; 12.22 ± 0.52) vs. 2 HC (all M; 12.64 ± 0.24)	Not provided	full-scale IQ ≥ 85 ADHD: 3 with ODD	5 ADHD stopped medications 2 days previous the experimental session	Laboratory recording with adaptation night; 2 EEG channels (C3, C4)	Macrostructure EEG power analysis at C3 (SWA; delta; theta; alpha; sigma, during REM and NREM sleep) Visual spindle detection in Stage 2	Children with ADHD had longer REM sleep duration and SOL than HC Children with. ADHD had shorter SWS latency and lower SE than HC. No significant differences on EEG power and spindle density were found between groups. Children with ADHD showed reduced sleep-associated consolidation of declarative memory. HC showed a correlation between sleep-associated recognition enhancement in declarative memory task (IAPS) and <1 Hz power during the first sleep cycle. NREM sleep duration in HC was positively correlated to sleep-related memory consolidation.
**Gruber et al. [28]**	26 ADHD (17 M; 8.61 ± 1.27) vs. 49 HC (30 M; 8.61 ± 1.27)	1 hyperactive 8 inattentive 17 combined	full-scale IQ ≥ 80 ADHD: 8 with ODD; 2 with conduct disorder	ADHD stopped medications 2 days previous the experimental session	Home recordings; 8 EEG channels (F3, F4, C3, C4, P3, P4, O1, O2)	Macrostructure	No significant differences were found on sleep parameters between groups.
**Kirov et al. [29]**	20 ADHD (19 M; 11.24 ± 2.31) vs. 19 HC (17 M; 11.26 ± 2.49)	All combined	full-scale IQ ≥ 80 None	11 ADHD stopped medications at least 7 days previous the experimental session	Laboratory recordings with adaptation night; 2 EEG channels (C3, C4)	Macrostructure	Children with ADHD had higher TBT, TST, shorter REM sleep latency and longer REM sleep duration than HC.
**Prihodova et al. [30] **	14 ADHD (12 M; 9.6 ± 1.6) vs. 12 HC (8 M; 9.0 ± 1.6)	2 inattentive 12 combined	IQ not specified, exclusion of mental retardation None	None	Laboratory recording with adaptation night; 4 bipolar EEG channels (F4-C4, C4-P4, F3-C3, C3-P3, C4-A1, C3-A2)	Macrostructure CAP analysis	No significant differences were found on sleep parameters between groups.
**Prehn-Kristensen et al. [31]**	16 ADHD (all M; 10.6 ± 0.95) vs. 16 HC (all M; 11.1 ± 0.95) vs. 20 HC adults (all M; 24.7 ± 2.8)	8 inattentive 8 combined	full-scale IQ ≥ 85 ADHD: 4 with ODD.	12 ADHD stopped medications 2 days previous the experimental session.	Laboratory recordings with adaptation night; 4 EEG channels (F3, F4, C3, C4)	Macrostructure EEG power analysis at F4. (SWA, delta and sigma during stage 2; theta during REM sleep)	No significant differences were found on sleep parameters between children groups. After merged all healthy subjects (children and adults), a correlation between emotional memory (investigated by IAPS) and slow/delta during SWS was found. ADHD showed negative correlation between performance and <1 Hz power during SWS. The same correlation was found with theta activity during REM sleep.
**Wiebe et al. [32]**	20 ADHD (13 M; 9.2 ± 1.6) vs. 46 HC (28 M; 8.8 ± 1.1)	3 hyperactive 13 inattentive 4 combined	mean IQ ADHD =100.4 mean IQ HC = 104.0 None	ADHD stopped medications at least 2 days previous the experimental session.	Home recordings; 8 EEG channels (F3, F4, C3, C4, P3, P4, O1, O2)	Macrostructure	No significant differences were found on sleep parameters between groups.
**Ringli et al. [33]**	9 ADHD (8 M; 11.9 range: 9.7–13.4) vs. 9 HC (8 M; 11.6 range: 9.6–14.2)	All combined	mean IQ 120±15 None	2 ADHD were treated at the day of experimental session. The second dose of medications was not given at the day of measurement.	Laboratory recording; High-density EEG (128 channels)	Macrostructure EEG power analysis in all cortical channels. (SWA during NREM sleep)	Children with ADHD had lower duration of stage 1 than HC. Children with ADHD showed higher SWA power over central than HC.
**Akinci et al. [34]**	28 ADHD (20 M; 10 range: 8–12) vs. 15 HC (9 M; 10 range: 9–13)	7 inattentive 21 hyperactive or combined	full-scale IQ > 70. None	None	Laboratory recordings with adaptation nigh; 10 EEG channels	Macrostructure CAP analysis	Children with ADHD had higher REM sleep duration than HC. Children with ADHD had lower total CAP rates than HC. Children with ADHD had a reduced total A1 index in stage 2.
**Virring et al. [35]**	76 ADHD (74% M; 9.6 ± 1.8) vs. 25 HC (68% M; 9.4 ± 1.5).	5 hyperactive 14 inattentive 57 combined	full-scale IQ > 70 ADHD: 6 with autism, 9 with internalizing comorbidity, 20 with externalizing comorbidity; 7 with tic disorder	None	Home recording; 6 EEG channels (F4, C4, O2, F3, C3, O1)	Macrostructure	Children with ADHD had higher numbers of sleep cycles, lower TST, lower stage 1 and 3 and longer REM sleep duration than HC. When children with comorbidity were excluded from the analyses, ADHD group showed only longer SOL than HC.
**Saletin et al. [36]**	7 ADHD (5 M; 11.9 ± 0.9) vs. 14 HC (10 M; 11.7 ± 0.9)	Not provided	mean IQ 110.3 ± 14.1. None	ADHD stopped medications 2 days previous the experimental session.	Laboratory recordings with adaptation night; 4 EEG channels (C3, C4, O1, O2)	Macrostructure EEG power analysis at C3, C4 (slow and fast sigma; SWA during Stage 2)	Children with ADHD had lower TBT than HC. Children with ADHD showed reduced sigma power (spindle-related) than HC. Children with ADHD showed lower MST before sleep than HC, but no overnight gain was observed. MST precision was positively associated with slow spindle activity for the children with ADHD.
**Cremone et al. [37] **	18 ADHD (13 M; 6.70 ± 1.07) vs. 15 HC (11 M; 6.73 ± 0.71)	All hyperactive	IQ not specified, exclusion of mental retardation None	ADHD stopped medications 2 days previous the experimental session	Laboratory recordings; 24 EEG channels	Macrostructure EEG power analysis in all cortical channels. (delta during stage 2 and SWS; theta, during REM and NREM sleep)	No significant differences were found on sleep parameters between groups. HC showed greater accuracy at go/noGo task in the morning vs. baseline after sleep. The performance was significantly associated with REM theta activity at F4. Children with ADHD showed greater theta activity in REM sleep than controls, however they revealed no changes in their performance after sleep.
**Wiesener et al. [38]**	17 ADHD (All M; 11.3 ± 0.4) vs. 17 HC (all M; 11.1 ± 0.2)	2 hyperactive 15 combined	full-scale IQ ≥ 85 ADHD: 14 with ODD, 3 with conduct disorder; 6 with learning disabilities.	13 ADHD stopped medications 2 days previous the experimental session	Laboratory recording with adaptation night; 2 EEG channels (C3, C4)	Macrostructure	No significant differences were found on sleep parameters between groups. Children with ADHD did not show sleep-dependent consolidation of rewarded behavior. Their consolidation of rewarded behavior did not correlate with sleep. Instead, HC consolidated rewarded behavior better during a night of sleep than during a day awake.
**Chin et al. [39]**	71 ADHD (54 M, 8.83 ± 1.86) vs. 30 HC (15 M, 8.48 ± 2.36)	35 inattentive 36 hyperactive or combined	full-scale IQ > 70 None	ADHD had no medications in the 6 months previous the experimental session.	Laboratory recordings; 32 EEG channels	Macrostructure	Children with ADHD had lower percentage of SWS and higher apnea-hypopnea index than HC.

ADHD, attention-deficit/hyperactivity disorder; IQ, intellectual quotient; PSG, polysomnographic; EEG, electroencephalographic; M, males; HC, healthy children; TBT, total bed time; SPT, sleep period time; REM, rapid eye movement; ODD, oppositional defiant disorder; CAP, cycling alternating pattern; TST, total sleep time; SS, stage shift; SWS, slow wave sleep; SE, sleep efficiency; PLMD, periodic limb movement disorder; SWA, slow wave activity; NREM, non-rapid eye movement; SOL, sleep onset latency; IAPS, international affective picture system; MST, motor sequence task.

## Supplementary Materials

The following are available online at https://www.mdpi.com/2077-0383/8/10/1737/s1, Table S1: List of abbreviations. The abbreviations including in the text are reported alphabetically.
