# Supplementary material for: Advances in Understanding the Relationship between Sleep and Attention Deficit-Hyperactivity Disorder (ADHD)
Source: J Clin Med. 2019 Oct 19;8(10):1737. doi: 10.3390/jcm8101737 (PMC6832299; doi:10.3390/jcm8101737)
Supplement: Supplementary file 1 [file jcm-08-01737-s001.pdf]

Table 1. List of abbreviations. The abbreviations including in the text are reported alphabetically.

Abbreviation	Full form	Definition
ADHD	Attention-Deficit Hyperactivity Disorder	ADHD is one of the most common early childhood disorders, classified into three subtypes: predominantly inattentive, hyperactive-impulsive, and a combination of these two subtypes.
CAP	Cycling Alternating Pattern	CAP is an EEG activity during NREM sleep that may represent an index of arousal and sleep instability.
DSM	Diagnostic And Statistical Manual Of Mental Disorders	DSM is one of the most used diagnostic manual by clinicians in the filed of mental health.
EEG	Electroencephalographic/Electroencephalography	EEG measures the electrical brain activity through electrodes placed on the scalp.
HC	Healthy Children	The group of HC the controls with a typical development represents in the mentioned studies.
IAPS	International Affective Picture System	IAPS is a database containing a standardized set of pictures for studying attention and emotion.
IQ	Intellectual Quotient	IQ is a score obtained from a standardized tests designed to evaluate the human intellectual functioning.
MSLT	Multiple Sleep-Onset Latency Test	MSLT is used to assess excessive sleepiness and diagnose narcolepsy. Subjects have a series of nap opportunities across the day.
MST	Motor Sequence Task	MST is designed to assess procedural skill learning. It required participants to tap a 5-element on the numeric keys of a keyboard with the fingers of their non-dominant hand "as quickly and accurately as possible" for 30 seconds. The task is administered at the evening and at the morning session after sleep.
NREM	Non Rapid Eye Movement	NREM sleep is divided into four stages in the Rechtschaffen and Kales standardization: stage 1, stage 2, stage 3 and stage 4.
ODD	Oppositional Defiant Disorder	ODD is a neurodevelopmental disorder characterized by frequent and persistent angry/irritable mood, argumentative/defiant behavior, or vindictiveness in children and adolescents. The disorder is often comorbid with ADHD.
PLMS/PLMD	Periodic Limb Movements Of Sleep/ Periodic Limb Movement Disorder	PLMS/PLMD is a disorder characterized by repetitive and involuntary movements in the lower limbs, that occur about every 20-40 seconds during sleep.
PSG	Polysomnographic/Polysomnography	PSG is a tool used to records brain waves and other parameters (e.g., eye movements, muscular tone, heart rate) during sleep. It is helpful to diagnose sleep abnormalities.
REM	Rapid Eye Movement	REM sleep is the stage characterized by rapid eye movements, frequent mental sleep activity and muscle atonia.
SDB	Sleep-Disordered Breathing	SDB refers to several chronic conditions in which partial or complete cessation of breathing occurs across the night.
SE	Sleep Efficiency	SE is the percentage of time spent asleep. It is represented by the ratio between the amount of total sleep time and the total time in bed. A SE of 85% or higher is considered to be normal.
SOL	Sleep Onset Latency	SOL is the duration of time it takes to fall asleep after the lights have been turned off/after "the goodnight".
SPT	Sleep Period Time	SPT is the amount of time from sleep onset to sleep end.
SS	Stage Shift	SS indicates a transition from a sleep stage to another.

		High rate of SS may result in complaints of non-restorative sleep.
SWA	Slow Wave Activity	SWA is an EEG synchronized activity ranged from0.5 to 4.0 Hz. In healthy people, it is maximally expressed during NREM sleep.
SWS	Slow Wave Sleep	SWS refers to the deep NREM sleep, consists of stage 3 and stage 4.
TBT	Total Bed Time	TBT represents the total time in bed.
TST	Total Sleep Time	TST is the amount of time spent asleep from sleep onset to the end of the final sleep epoch. Differently from SPT, this index does not include the wakefulness after sleep onset.
WASO	Wakefulness After Sleep Onset	WASO is the amount of time spent awake, starting from the sleep onset to the last awakening.
